# Broadband infrared study of pressure-tunable Fano resonance and metallization transition in 2H-$\hbox {MoTe}_2$

**DOI:** 10.1038/s41598-022-22089-0

**Published:** 2022-10-15

**Authors:** E. Stellino, F. Capitani, F. Ripanti, M. Verseils, C. Petrillo, P. Dore, P. Postorino

**Affiliations:** 1 Department of Physics and Geology, University of Perugia, via Alessandro Pascoli, 06123 Perugia, Italy; 2 Synchrotron SOLEIL, L’Orme des Merisiers, 91190 Saint-Aubin, Gif-sur-Yvette France; 3 Sapienza University, Piazzale Aldo Moro, 2, 00185 Rome, Italy

**Keywords:** Physics, Condensed-matter physics, Semiconductors, Two-dimensional materials

## Abstract

High pressure is a proven effective tool for modulating inter-layer interactions in semiconducting transition metal dichalcogenides, which leads to significant band structure changes. Here, we present an extended infrared study of the pressure-induced semiconductor-to-metal transition in 2H-$\hbox {MoTe}_2$, which reveals that the metallization process at 13–15 GPa is not associated with the indirect band-gap closure, occurring at 24 GPa. A coherent picture is drawn where n-type doping levels just below the conduction band minimum play a crucial role in the early metallization transition. Doping levels are also responsible for the asymmetric Fano line-shape of the $\hbox {E}_{1u}$ infrared-active mode, which has been here detected and analyzed for the first time in a transition metal dichalcogenide compound. The pressure evolution of the phonon profile under pressure shows a symmetrization in the 13–15 GPa pressure range, which occurs simultaneously with the metallization and confirms the scenario proposed for the high pressure behaviour of 2H-$\hbox {MoTe}_2$.

## Introduction

Transition Metal Dichalcogenides (TMDs) are layered crystals that exhibit a graphene-like lattice structure, with strong intra-layer covalent bonds and weak van der Waals forces between adjacent planes. Semiconducting TMDs have band gaps that are tunable with the number of layers^1–3^ and span in the range of 1–2.5 eV depending on the crystal composition. In particular, 2H-$\hbox {MoTe}_2$ (hereinafter $\hbox {MoTe}_2$) displays an indirect band-gap $\hbox {E}_g^I\sim$ 0.9 eV and a direct gap $\hbox {E}_g^D \sim \, 1.1$ eV^4^, which make it one of the few TMDs exploitable for optoelectronic devices in the near-infrared range^5–7^.

Tuning the electronic properties through the modulation of the inter-layer interaction can further boost the technological applications for $\hbox {MoTe}_2$ crystals. This can be achieved by changing the number of layers of the sample, or, alternatively, by applying high pressure, which allows for an analysis of the band structure modifications when the inter-layer bonds strengthen.

Previous studies based on DFT calculations have shown that the $\hbox {MoTe}_2$ lattice symmetry is maintained up to 50 GPa^8^, in agreement with high-pressure Raman and X-Rays diffraction measurements^9–12^ carried out up to $\sim$ 30 GPa. However, the compression of the inter-layer distance causes an increase in the inter-layer coupling that drives the modification of the electronic band structure, in analogy with what was observed for other TMD semiconductors^13,14^. In particular, theoretical and experimental works have shown the occurrence of a pressure-induced metallization, although the definition of a specific pressure range for the onset of the metallic phase is still controversial. In particular, DFT calculations by Riflikova et al. placed the transition in the range 13–19 GPa^15^. The Raman measurements of Bera et al. suggested an electronic transition around 6 GPa^10^. Finally, recent resistivity measurements provided evidence for the metallization above $\sim \,10$ GPa^9^ and, specifically, at $\sim \,15$ GPa^11^. In these two papers, DFT calculations were also performed, showing a band-gap closure at $\sim \,10$ GPa^9^ and $\sim \,13$ GPa^11^. Although all the above-mentioned works implicitly associated the onset of the metallization process with the indirect band-gap closure, none of them provided a direct experimental determination of the $\hbox {E}_g^I$ pressure dependence.

Within this still debated picture, the role played by intrinsic doping in the metallization process has never been adequately considered, although experimental evidence indicates that defect-related intra-gap levels strongly contribute to the optical and electronic response of semiconducting TMDs^16,17^. Photoluminescence (PL) spectra of few-layers compounds exhibit excitonic bands ascribable to radiative recombination processes involving doping states^18–20^, and resistivity measurements show that these extra levels significantly modify the transport properties of the samples, even when the doping is *unintentional* and, thus, the correspondent carrier density very low^17^.

In this perspective, the possibility of studying the pressure evolution of the doping levels and its contribution to the metallization process in TMDs might represent the missing piece for a complete understanding of the semiconductor-to-metal transition in these materials. However, addressing this investigation in bulk samples with low percentages of defects is far from a trivial task. Although doping-related exciton bands are well visible in the PL spectra of few-layer flakes at ambient conditions, their intensity strongly reduces in bulk samples. Moreover, above a few GPa, the increase in non-radiative recombination processes completely suppresses the photoluminescence signal of TMDs^21,22^, preventing studying the exciton response approaching the metallization.

We will show, here, that synchrotron-based infrared spectroscopy can provide us with an unmatched tool to decouple the different, inter-playing aspects driving the pressure evolution of the $\hbox {MoTe}_2$ electronic properties. While measurements in the near-infrared allow following the trend of the *standard* electronic transitions as a function of pressure, a careful investigation of the far infrared spectrum can give us information about the fundamental contribution from doping-related excitations^23^. Indeed, we found that the phonon spectrum of $\hbox {MoTe}_2$ exhibits a strong Fano resonance, which can be exclusively attributed to the coupling between the $\hbox {E}_{1u}$ mode and low-energy electronic transitions from doping levels at the bottom of the conduction band minimum. The application of pressure proved itself an effective tool to modulate the resonance, which actually disappears in correspondence with the onset of the metallic behavior. In this sense, the evolution of the Fano profile provides us with a unique benchmark to evaluate the role of the doping levels in the metallization process.

The observed phenomenology shares similarities with the gate-tunable Fano resonance observed in the mid infrared spectrum of bilayer graphene, where the application of high-voltage generates a band gap responsible for small-energy electronic transitions coupled with the G phonon mode^24^. To the best of our knowledge, the one we here report represents the first study of a pressure-modulated Fano resonance in TMDs and could definitively open the route to the exploration of new physical phenomena in 2D materials.

## Methods

A $\hbox {MoTe}_2$ crystal, provided by HQ Graphene, was exfoliated to obtain fresh-cut samples. Room temperature (RT), high-pressure infrared transmission measurements were carried out at the SOLEIL synchrotron. 0–27 GPa pressure range was achieved by using a Diamond Anvil Cell (DAC). Diamonds with culets of $400\ \upmu \hbox {m}$ were separated by a pre-indented stainless steel $50\ \upmu \hbox {m}$ thick gasket, in which a hole of $150\ \upmu \hbox {m}$ diameter was drilled. The exfoliated sample was positioned in the hole together with the pressure transmitting medium and a ruby chip, to measure the pressure through the ruby fluorescence technique. The measured samples typically had a 50 $\times$ 50 $\upmu \hbox {m}^2$ surface and micrometric thickness (bulk crystals).

NIR measurements were performed over the 4500–9000 cm$^{-1}$ range at the SMIS beamline, using a Thermo Fisher iS50 interferometer equipped with a quartz beamsplitter. Synchrotron edge radiation was employed as a light source. Custom-made 15$\times$ Cassegrain objectives with a large working distance allowed focusing and then collecting the transmitted radiation, which was finally detected by a liquid-nitrogen cooled mercury–cadmium–telluride detector^25^. NaCl powder was used as a quasi-hydrostatic medium^26^.

FIR measurements were carried out by using the setup available at the AILES beamline^27,28^. The Bruker IFS 125 HR interferometer, coupled with the synchrotron source, was equipped with a multi-layer $6\ \upmu \hbox {m}$ beamsplitter and a 4K-bolometer. Polyethylene powder was used as a pressure transmitting medium^29^ allowing for reliable measurements over the 100–600 $\hbox {cm}^{-1}$ spectral range.

In both FIR and NIR experiments, the absorbance spectrum $A({\tilde{\nu }})=-ln[I({\tilde{\nu }})/I_0({\tilde{\nu }})]$ was obtained at each pressure, with $I({\tilde{\nu }})$ the intensity transmitted with the $\hbox {MoTe}_2$ sample loaded in the cell and $I_0({\tilde{\nu }})$ the background intensity with the DAC filled by the hydrostatic medium only.

Finally, ambient condition measurements in the FIR range were performed by a Nicolet NicPlan microscope (32$\times$ magnification, 100 $\times$ 100 $\upmu$m$^2$ area) coupled with synchrotron radiation as a light source and a liquid He bolometer as a detector. In this case, larger samples with $\sim$ 200 $\times$ 200 $\upmu$m$^2$ area were measured.

## Results

### NIR measurements

Room temperature NIR transmission spectra were collected over the 0–17 GPa and 4–27 GPa pressure ranges on two $\hbox {MoTe}_2$ samples with different thickness: $\sim \,3.3$ $\upmu \hbox {m}$ (1st sample) and $\sim \, 1.3$ $\upmu \hbox {m}$ (2nd sample). Raw transmission spectra, reported in Fig. S1 of ***Supplemental Material (SM), show clear interference fringes at low pressure whose amplitude decreases as pressure and absorption increase (see ***Fig. S2 and the related discussion in the second section of SM). Applying a simple Fourier spectral smoothing procedure, the interference fringes are consistently reduced, emphasizing the sample absorption profile.

**Figure 1:**
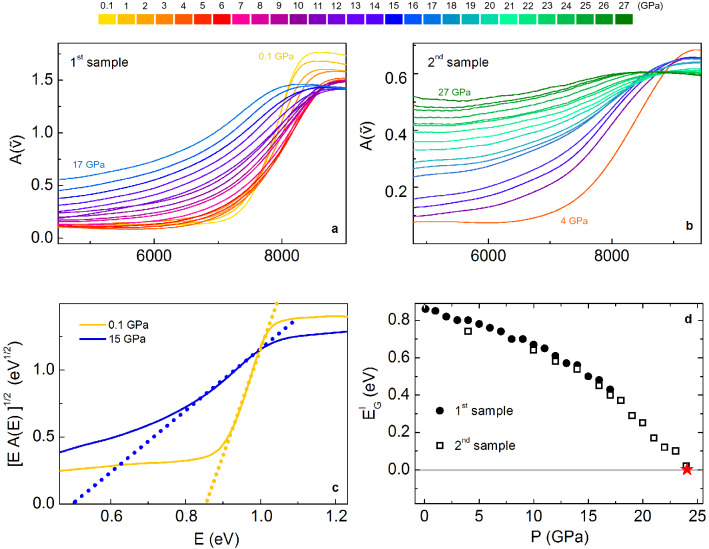
(**a**) Absorbance spectra collected in the first run over the 0.1–17 GPa range. (**b**) Absorbance spectra collected in the second run over the 4–27 GPa range. (**c**) Tauc plot at P = 0.1 GPa and P = 15 GPa and linear extrapolation (dotted lines) of the correspondent gap energies. (**d**) Energy gap as a function of pressure for the first (dots) and the second (open squares) run. The red star indicates the gap closing pressure, P $\sim$ 24 GPa.

The absorbance spectra $A({\tilde{\nu }})$, obtained after the Fourier smoothing procedure, are shown in Fig. 1a,b for the 1st and the 2nd sample, respectively. The absorbance exhibits a sigmoid shape, which is well defined at P = 0.1 GPa and progressively flattens as the pressure increases. The inflexion point of the sigmoid curve shifts rather continuously towards the low-frequency side of the spectrum.

This behavior is related to the pressure evolution of $\hbox {MoTe}_2$ electronic properties. Indeed, the steep rise of the absorbance at high frequencies (above $\sim$ 7000 $\hbox {cm}^{-1}$) can be ascribed to electronic transitions from valence to conduction band^30–32^. As mentioned in the introduction, in $\hbox {MoTe}_2$ both the direct, at $\sim \, 1.1$ eV, and the indirect, $\sim$ 0.9 eV, band-gaps^4^ lie within the NIR energy range here explored and the energy difference between the two gaps is $\sim$ 0.2 eV ($\sim$ 1600 $\hbox {cm}^{-1}$), significantly smaller than in other TMD semiconductors. In principle, both transitions could be visible in the measured spectra. However, as already observed by Lezama et al.^31^, when the crystal thickness is larger than 1 $\upmu \hbox {m}$, the high absorption due to the indirect transition strongly reduces the transmitted intensity above $\hbox {E}_g^I$, preventing the observation of the direct optical transition at the higher energy $\hbox {E}_g^D$. We also note that, in the present case, the transmitted intensity is further reduced by the reflectivity of the diamond anvil's faces, making even more difficult to reveal the signature of the direct transition. Since the thickness of our samples is larger than $1\, \upmu \hbox {m}$, we can safely ascribe the steep rise observed in $A({\tilde{\nu }})$ above 7000 $\hbox {cm}^{-1}$ to the indirect-gap transition.

In the case of indirect gap transition, the absorption coefficient $\alpha ({\tilde{\nu }})$ in the neighborhood of $\hbox {E}_g^I$ can be written as: $\sqrt{\alpha ({\tilde{\nu }})\cdot \hbar {\tilde{\nu }}}\propto \hbar {\tilde{\nu }}-E_g^I \pm E_{ph}$, where $E_{ph}$ is the energy of the phonon required by the momentum conservation. Under the reliable assumption that the measured $A({\tilde{\nu }})$ is simply proportional to $\alpha ({\tilde{\nu }})$ and neglecting the phonon energy $E_{ph}$, $\hbox {E}_g^I$ can be determined by using the so-called Tauc plot. This amounts to exploiting a linear regression for the quantity $\sqrt{\alpha ({\tilde{\nu }})\cdot \hbar {\tilde{\nu }}}$^32–34^, as shown in Fig. 1c. The Tauc plot procedure was applied to all the experimental absorption spectra (more details in the third section of SM). Using this procedure, the energy gap at 0.1 GPa is 0.86 eV, in perfect agreement with the 0.88 ± 0.05 eV value recently observed in infrared measurements at ambient conditions^31^. The resulting $\hbox {E}^I_g$ values are shown in Fig. 1d as a function of pressure for both the considered samples. The two data sets are in good agreement over the common pressure region and show a progressive reduction of the band-gap that finally closes at P $\sim$ 24 GPa. As the pressure applied on the sample is released, the band-gap progressively re-opens and the original band structure can be recovered once the sample is brought back to ambient conditions; see ***Fig. S12 in the SM.

Interestingly, the gap closing pressure obtained through the Tauc plot extrapolation in the NIR is significantly higher with respect to the metallization threshold already obtained by resistivity measurements^2,9^. This observation suggests a complex scenario for the evolution of the $\hbox {MoTe}_2$ electronic properties, which might comprise distinct charge delocalization processes occurring at different pressure values (discussed in next sections).

### FIR measurements

**Figure 2:**
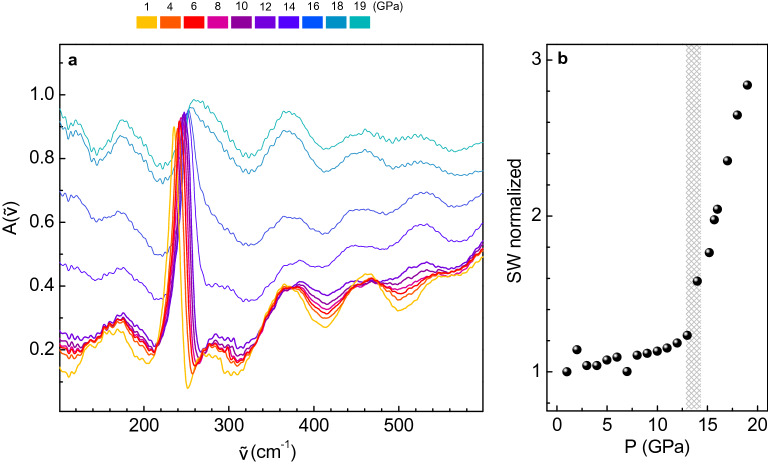
(**a**) Absorbance spectra at selected pressure values over the 1–19 GPa range. (**b**) Normalized spectral weight $SW(P,P_0)$ (see text); the vertical stripe highlights the pressure range where the change of slope occurs.

Since, based on our previous results, the $\hbox {MoTe}_2$ metallic behavior observed in the literature around 10–15 GPa cannot be ascribed to the pressure evolution of the high-energy electronic transitions, we investigated the pressure response of the low-energy contributions through the analysis of the $A({\tilde{\nu }})$ spectra in the FIR region over the 1–19 GPa range (Fig. 2a). All the measured $A({\tilde{\nu }})$ curves exhibit well visible oscillating fringes, which arise from interference effects between the diamond faces through the polyethylene medium in the background spectrum, as discussed in the fourth section of SM. Although a simple Fourier spectral smoothing procedure was not effective to remove the interference fringes, a sharp peak at about 235 $\hbox {cm}^{-1}$ at low pressure was quite apparent and it was assigned, according to the literature^35,36^, to the $\hbox {E}_{1u}$ phonon. The phonon peak also shows a clear broadening and a frequency hardening on increasing pressure, an effect that will be discussed in detail in the next section. Focusing on the overall trend of the spectra reported in Fig. 2a, it can be noticed that they remain almost overlapped at low pressures (approximately over 0–13 GPa), while their intensities rapidly rise on further increasing pressure. A simple application of Drude model proves that in the low-frequency limit, i.e. ${\tilde{\nu }}/\Gamma \ll 1$ with $\Gamma =c/\tau$ and $\tau$ Drude relaxation time, the experimental absorbance is simply proportional to the square root of both the DC conductivity $\sigma _0$ and the frequency ${\tilde{\nu }}$, thus resulting $A({\tilde{\nu }}) \propto \sqrt{\sigma _0 {\tilde{\nu }}}$. Therefore, the significant growth in the overall absorption at high pressure can be related to an increase of $\sigma _0$^37^ and, thus, to the onset of a metallic behavior. To carry out a quantitative analysis, we define the absorption spectral weight at each pressure: $\displaystyle {sw(P)=\int _{{\tilde{\nu }}_m}^{{\tilde{\nu }}_M}{A({\tilde{\nu }})d{\tilde{\nu }}}}$. We then conveniently introduce the normalized quantity $SW(P,P_0)= sw(P)/sw(P_0)$, which describes the variation of the spectral weight from the minimum pressure value $\hbox {P}_0 =1$ GPa. The $SW(P,P_0)$ values obtained with ${\tilde{\nu }}_m=100\,\hbox { cm}^{-1}$ and ${\tilde{\nu }}_M=600\,\hbox { cm}^{-1}$ are shown in Fig. 2b. The integration does not include the 200–270 $\hbox {cm}^{-1}$ range where the phonon contribution is dominant (although including the phonon frequency region or varying the ${\tilde{\nu }}_m$ and ${\tilde{\nu }}_M$ integration limits does not appreciably modify the overall trend). Our results indicate the onset of a phase with metallic characteristics between 13 and 15 GPa, in good agreement with previous resistivity measurements^9,11^. It is worth underlining that the metallic behaviour can be reversed once the pressure on the sample is released, as shown in ***Fig. S12 of the SM, coherently with what was reported by Yang et al.^11^.

### The Fano resonance

**Figure 3:**
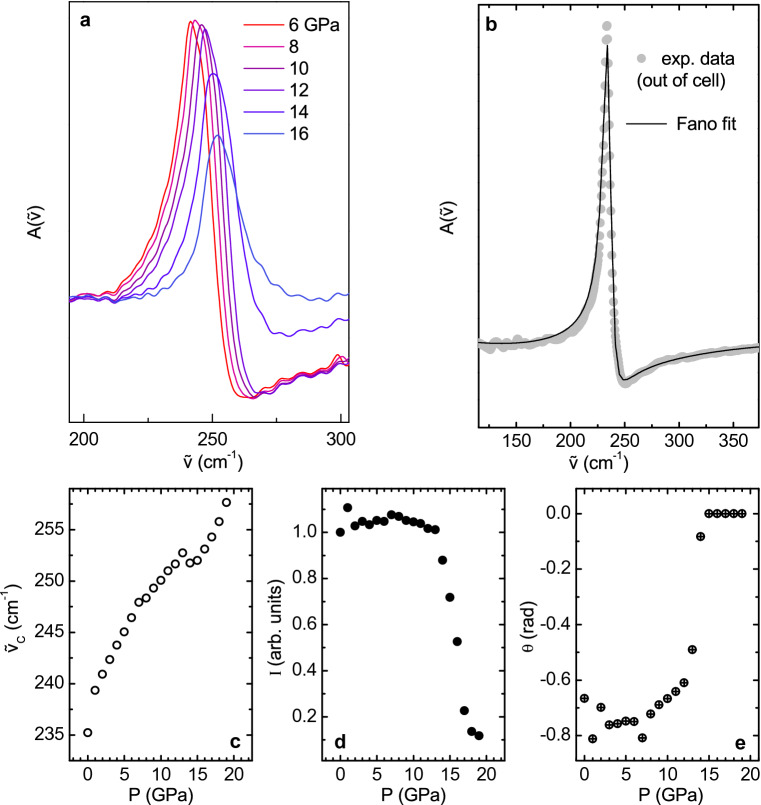
(**a**) Absorbance spectra at selected pressures after the subtraction of a parabolic background and of sinusoidal fringes. (**b**) Experimental data at ambient pressure (dots) fitted by a Fano function (continuous line): $\displaystyle {F({\tilde{\nu }})=F\cdot \frac{{\tilde{\nu }}^2\cos \theta +{\tilde{\nu }}({\tilde{\nu }}^2-{\tilde{\nu }}_C^2)\sin \theta }{{\tilde{\nu }}^2\gamma ^2+({\tilde{\nu }}^2-{\tilde{\nu }}_C^2)} }$. (**c**) $\hbox {E}_{1u}$ peak position ${\tilde{\nu }}_C$ as a function of pressure. (**d**) Peak intensity as a function of pressure, normalized to the 1 GPa value. (**e**) $\theta$ parameter of the Fano fit as a function of pressure.

As mentioned, FIR absorption spectra exhibit an intense phonon peak at 235 $\hbox {cm}^{-1}$ attributed to the $\hbox {E}_{1u}$ vibrational mode. A close inspection of the raw data shown in Fig. 2a reveals that the $\hbox {E}_{1u}$ peak presents an unusual asymmetric shape in the low pressure regime, and it is notably affected by pressure. To rule out the effect of the diamond-diamond interference fringes on the line-shape asymmetry, a further absorption measurement was carried out on a free-standing $\hbox {MoTe}_2$ slab (i.e. at P = 0 GPa and without interference effects). The line-shape of the out-of-cell phonon peak, shown in Fig. 3b, displays a remarkable Fano-like profile^38^ in close similarity with that observed in the inside-cell measurements (a direct comparison between the in-cell and out-of-cell profiles is reported in ***Fig. S11 of SM).

The measured phonon peak line-shape was modelled by using a Fano-profile^39^ with a second-order polynomial to fit the background. The Fano profile is given by:

1 $$\begin{aligned} F({\tilde{\nu }})=F\cdot \frac{{\tilde{\nu }}^2\cos \theta +{\tilde{\nu }}({\tilde{\nu }}^2-{\tilde{\nu }}_C^2)\sin \theta }{{\tilde{\nu }}^2\gamma ^2+({\tilde{\nu }}^2-{\tilde{\nu }}_C^2)} \end{aligned}$$

where ${\tilde{\nu }}_C$ is the peak centre, $\gamma$ is the peak width, and $\theta$ is the phase associated with the line asymmetry. Notice that $\theta$ is directly related to the well-known Fano parameter *q* by $\tan (\theta /2)=1/q$^38,39^ with, however, the numerical advantage of avoiding the *q* divergence. The symmetric Lorentzian profile, corresponding to the non-resonance condition, is indeed recovered when $\theta =0$ (i.e. $q \rightarrow \infty$). In Fig. 3b, we can appreciate the very good agreement between the best fit curve and the experimental data at P = 0 GPa.

This result validates a fitting procedure of the pressure-dependent spectra based on the combination of three functions: a second-order polynomial background, a Fano-profile for the $\hbox {E}_{1u}$ phonon, and a sinusoidal curve for the interference fringes (more details in the fifth section of SM). Spectra collected at selected pressure values after electronic background and fringes subtraction are shown in Fig. 3a.

The best fit peak parameters of the Fano profiles are shown in Fig. 3c–e as a function of pressure. The peak central frequency ${\tilde{\nu }}_C$ (Fig. 3c) shifts toward higher values on increasing pressure, as expected. Therefore, the weak anomaly observed at $\sim$13 GPa in the pressure dependence of the peak frequency ${\tilde{\nu }}_C$ could be ascribed to some electronic effects. Correspondingly, the $\hbox {E}_{1u}$ intensity (Fig. 3d) remains almost constant up to 13 GPa and then rapidly decreases. This is interpreted as a consequence of the optical response of free electrons that screen the phonon contribution in the FIR absorption. As to the peak profile, Fig. 3e clearly shows that the $\theta$ parameter keeps the almost constant value $\sim -$ 0.7 rad up to 13 GPa, and then abruptly goes to zero as the peak symmetrizes and the Fano coupling disappears. The overall line-shape analysis thus consistently points out the presence of a pressure threshold of electronic nature above 13 GPa.

The Fano theory^39^ explains the asymmetric profile of a given discrete excitation as a consequence of the coupling between the excitation itself and a continuum of excited states with the same energy scale. In the present case, the discrete state is undoubtedly the $\hbox {E}_{1u}$ phonon, with energy $\hbox {E}_{ph}=0.03$ eV, whereas the continuum needs to be identified by some suitable electronic transitions with the appropriate energy. The $\hbox {MoTe}_2$ band-gap is $\hbox {E}_g$ $\sim$ 0.9 eV, which is more than one order of magnitude larger than $\hbox {E}_{ph}$. The phonon coupling with a transition from valence to conduction band, thus, cannot realistically take place. However, since the measured samples are n-type semiconductors, as declared by the manufacturer HQ-Graphene and like most of the TMDs grown by Chemical Vapor Transport^9,17^, charges in excess giving rise to extra electronic levels, just below the conduction band minimum, could be responsible for electronic excitations with energies comparable with $\hbox {E}_{ph}$, as also reported in Ref.^40^. It is worth noticing that a negative asymmetric parameter $\theta$, as obtained from the experimental data fit, is perfectly consistent with the presence of n-type doping that is known to cause an asymmetric broadening of the peak on the lower energy side, as previously observed for few-layer graphene^41–43^. Conversely, p-type doping would have produced hole states in the proximity of the valence band maximum and an asymmetric broadening of the peak on the higher energy side.

**Figure 4:**
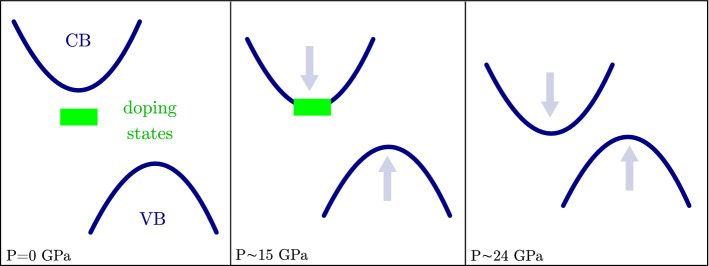
Graphic illustration of the two-step mechanism that we proposed to describe the pressure-induced metallization process in $\hbox {MoTe}_2$. At ambient pressure (P = 0 GPa), the n-type doping levels lie just below the conduction band minimum and the indirect gap energy is $\sim$ 0.9 eV. At P $\sim$ 13 to 15 GPa, we hypothesize an overlap between doping levels and conduction band, witnessed by the symmetrization of the Fano resonance, which is responsible for the metallic behavior of $\hbox {MoTe}_2$ observed in both FIR and transport measurements; in this configuration, however, the indirect gap is still open. Finally, at $\hbox {P}\sim$ 24 GPa, the indirect gap closes.

In the present scenario, the pressure evolution of the phonon lineshape can be consistently explained: indeed, as the pressure increases, the energy separation between the conduction band minimum and the doping levels progressively reduces, until vanishing above 13–15 GPa; see Fig. 4. This process not only is responsible for the increase in the absorption spectral weight observed in the FIR in the high-pressure regime, but it also causes the observed phonon peak symmetrization. Indeed, when the energy gap between the doping level and the conduction band goes to zero, no more direct electronic transitions from inter-band states are available for the phonon to couple with, preventing the existence of the Fano resonance in the $\hbox {E}_{1u}$ profile.

As previously mentioned in the introduction, the phenomenology and the interpretation here proposed for the pressure evolution of the Fano resonance in $\hbox {MoTe}_2$ shares analogies with the gate-tunable Fano resonance observed in the G mode of graphene^24^. In that case, the application of high voltage opened a finite band gap, allowing for electronic excitations coupled with the phonon mode. Here, on the other hand, the application of pressure drives the closure of the energy separation between doping levels and conduction band, suppressing the direct electronic transitions coupled with the $\hbox {E}_{1u}$ mode and, thus, the resonance in the metallic regime. In both cases, we achieve tuning of the electron–phonon coupling through the external modulation of the doping states in the sample.

## Discussion

Our results from the different series of IR measurements at low (FIR) and high (NIR) frequency enabled a deeper understanding of the metallization process in bulk $\hbox {MoTe}_2$, unravelling the mechanisms that drive the transition and the role played by the doping levels. The interpretation model we propose largely reconciles the apparently controversial results present in the literature on the metallization transition in $\hbox {MoTe}_2$, where the metallization pressure was reported to vary from 6^10^ to $\sim$ 20 GPa^15^. Our experiment reveals that the spectroscopic signatures of the metallization are related to different microscopic mechanisms, which allow framing the electronic transitions in a more complex scenario.

The NIR measurements enabled us to identify the indirect nature of the transition and to obtain the band-gap energy as a function of pressure. The pressure dependence of the energy gap revealed the closure of the band-gap at pressures above $\sim$ 24 GPa, a value rather close to the upper limit of the pressure range indicated by the most detailed DFT calculations by Riflikova et al.^15^. Those DFT calculations focused on the study of the pressure behaviour of the electronic bands, identifying the occurrence of the metallization when the band overlap occurs. It is worth noticing that the effect of doping is not accounted for in DFT calculations and it is not detectable in the NIR experiment.

In the FIR region, the spectral weight analysis of the measured absorbance showed a slowly raising trend at low pressures followed by an abrupt increase at 13–15 GPa. This behavior can be consistently explained by considering the pressure evolution of the doping levels in the band structure. We know that, at ambient temperature, the doping electrons can be thermally excited to the conduction band with a probability that depends upon the energy distance between the doping and the conduction band states ($\sim \, 0.03$ eV at ambient pressure). Based on our hypothesis, on applying pressure, this energy distance reduces, leading to a progressive increase in the thermally excited electrons in the conduction band and, thus, to a small, still progressive, increase in the absorption spectral weight *SW* in the range 0–13 GPa. Following the interpretation discussed in the previous section, we believe that above 13–15 GPa a band crossing occurs between the doping levels and the conduction band; see Fig. 4. As a consequence, dopant electrons are no longer bound to impurity sites and new conduction-band-like states are available for charge transport without thermal activation. In this situation, we observe an abrupt increase in the absorption spectral weight, which arises from the delocalization of the dopant electrons due to the hybridization of defect states with the conduction band^44^.

This finding is in very good agreement with the results from resistivity measurements reported in the literature^9,11^, which fix the onset of the metallization in the 10–15 GPa pressure range.

The relevance of the doping levels in the low energy electrodynamics is strongly witnessed by the Fano line-shape, here observed for the first time in the $\hbox {E}_{1u}$ peak of $\hbox {MoTe}_2$. The occurrence of a Fano resonance indirectly provides an estimate for the energy gap between the conduction band minimum and the doping level in the band structure, which should be of the same order of magnitude as the $\hbox {E}_{1u}$ phonon, i.e. $\sim$ 0.03 eV. Moreover, the pressure evolution of the phonon line-shape towards a symmetric Lorentzian profile at high pressure is consistent with the closure of the small energy gap exciton-conduction band. According to the Fano theory, the symmetrization of the $\hbox {E}_{1u}$ peak should occur when all the direct electronic transitions with energy comparable to $\hbox {E}_{ph}$ are suppressed. The peak symmetrization and the metallization onset are indeed observed at the same pressure.

In conclusion, our work provides the first reliable measurement of the $\hbox {MoTe}_2$ indirect band-gap energy as a function of pressure, indicating a gap-closing pressure above 24 GPa. We are able to provide an explanation for the early metallization observed in both FIR and electrical resistivity measurements by taking into account the doping effects. The pressure evolution of the Fano resonance in the $\hbox {E}_{1u}$ peak, here observed and interpreted for the first time, strongly supports the key role played by the doping levels in determining the sample electronic properties and sheds light on the pressure-induced modulation of the electron-phonon coupling in TMDs. We finally underline that the present measurements clearly demonstrated the capability of infrared spectroscopy to provide deeper understanding of the electronic properties of TMDs either at ambient conditions or under high applied pressure.

## Supplementary Information

Supplementary Information.

## Data Availability

The data that support the findings of this study are available from the corresponding author upon reasonable request.
