# Supplementary material for: Broadband infrared study of pressure-tunable Fano resonance and metallization transition in 2H-MoTe2
Source: Sci Rep. 2022 Oct 15;12:17333. doi: 10.1038/s41598-022-22089-0 (PMC9569381; doi:10.1038/s41598-022-22089-0)
Supplement: Supplementary file 1 — Supplementary Information. [file 41598_2022_22089_MOESM1_ESM.pdf]

Supplemental Material: Broadband Infrared Study of Pressure-Tunable Fano Resonance and Metallization Transition in 2H-$MoTe_2$.

E. Stellino,*,† F. Capitani,*,‡ F. Ripanti,† M. Verseils,‡ C. Petrillo,† P. Dore,¶ and P. Postorino¶

†*Department of Physics and Geology, University of Perugia, via Alessandro Pascoli, Perugia, Italy*

‡*Synchrotron SOLEIL, L'Orme des Merisiers, Saint-Aubin, Gif-sur-Yvette, France*

¶*Department of Physics, Sapienza University of Rome, P.le A. Moro 5, Rome, Italy*

E-mail: elena.stellino@studenti.unipg.it; francesco.capitani@synchrotron-soleil.fr

1 - Raw spectra in the near infrared range

Absorbance spectra are defined as

$$A(\tilde{\nu}) = -log\frac{I_T^{Smpl}(\tilde{\nu})}{I_T^{Bkg}(\tilde{\nu})}$$

where I_T^{Bkg} is the intensity transmitted by the DAC filled with NaCl only and I_T^{Smpl} is the intensity transmitted by the DAC with the $MoTe_2$ sample on top of NaCl. Raw absorbance spectra collected on the 1st and 2nd sample are reported in Fig. S1 (a), (b) respectively.

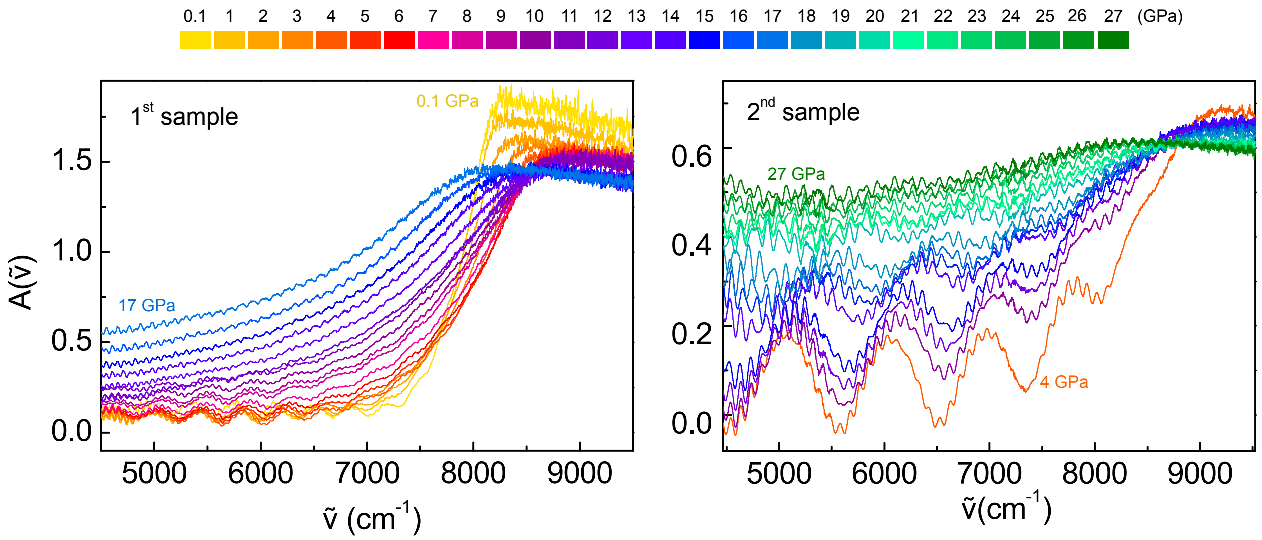

Figure S1: (a) Raw absorbance spectra collected on the 1st sample over the 0.1-17 GPa range. (b) Raw absorbance spectra collected on the 2nd sample over the 4-27 GPa range. .

2 - Interference Fringes in the NIR range

Fig. S2 shows a selection of spectra of the transmitted intensity collected for the 1st sample (see main text), namely $I_T^{Bkg1}(\tilde{\nu})$, $I_T^{Smpl1}(\tilde{\nu})\Big|_{P=0.1GPa}$, $I_T^{Smpl1}(\tilde{\nu})\Big|_{P=17GPa}$.
$I_T^{Bkg1}(\tilde{\nu})$ exhibits well-visible interference fringes with period $\Omega_1 \sim 55$ cm^{-1}. These oscillations can be attributed to interference effects due to multiple reflections of the incident beam between the diamond faces through the NaCl medium.
$I_T^{Smpl1}(\tilde{\nu})\Big|_{P=0.1GPa}$ is characterized by the presence of two sets of fringes with different period, as shown in Fig. S2. The oscillation with smaller amplitude and period is very similar in both period and phase to the one observed in $I_T^{Bkg1}(\tilde{\nu})$. This is particularly clear in the low-wavenumber region where the sample absorption is not high enough to prevent the reflection of the incident beam between the diamond culets. The second oscillation with period $\Omega_1' \sim 380\ cm^{-1}$, instead, can be attributed to interference effects between the surfaces of the thin sample slab. On increasing pressure (see the $I_T^{Smpl1}(\tilde{\nu})\Big|_{P=17GPa}$ spectrum in fig. S2), the sample absorption increases and the amplitude of both oscillations significantly reduces, as expected.

Since the fringe spacing can be simply related to the sample refractive index and thickness,

if we assume $n_{NaCl} \sim 1.5$[1] and $n_{MoTe_2} \sim 4$,[2] we get ~60 μm for the distance between the diamond culets and ~3.3 μm for the thickness of the 1st sample.

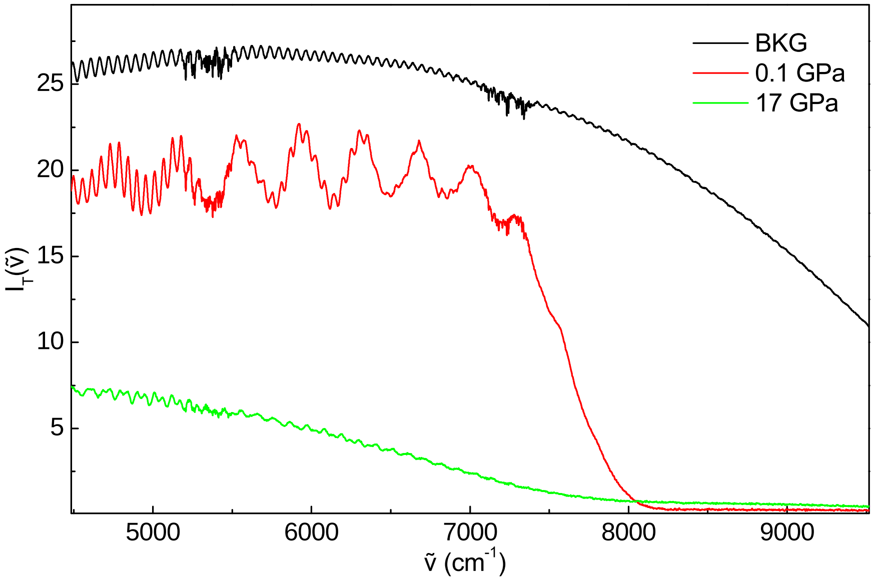

Figure S2: Spectra of the transmitted intensity collected on the background and on the 1st sample at P=0.1 GPa and P=17 GPa.

The same argument can be applied to the 2nd sample. Figure S3 shows a selection of spectra of the transmitted intensity collected for the 2nd sample (see main text), namely $I_T^{Bkg2}(\tilde{\nu})$, $I_T^{Smpl2}(\tilde{\nu})\Big|_{P=4GPa}$, $I_T^{Smpl2}(\tilde{\nu})\Big|_{P=24GPa}$.

$I_T^{Bkg2}(\tilde{\nu})$ exhibits well-visible interference fringes with period $\Omega_2 \sim 55$ cm^{-1}. As in the previous case, these oscillations can be attributed to interference effects due to multiple reflections of the incident beam between the diamond faces through the NaCl medium.

$I_T^{Smpl2}(\tilde{\nu})\Big|_{P=4GPa}$ is characterized by the presence of two sets of fringes with different periods. The oscillation with smaller amplitude and period is very similar in both period and phase to the one observed in $I_T^{Bkg2}(\tilde{\nu})$. The second oscillation (period $\Omega_2' \sim 1000$ cm^{-1}), instead, can be attributed to interference effects between the the surfaces of the thin sample slab. On increasing pressure, the sample absorption increases and the amplitude of both oscillations significantly reduces, as reported in Fig. S3.

In analogy to the previous case, the oscillation period in $I_T^{Bkg2}(\tilde{\nu})$ ($\Omega_2 \sim 55$ cm^{-1}) and $I_T^{Smpl2}(\tilde{\nu})\Big|_{P=4GPa}$ ($\Omega_2' \sim 1000$ cm^{-1}) can be used to estimate the distance between the dia-

mond culets, $\sim 60\ cm^{-1}$, and the 2nd $MoTe_2$ sample thickness, $\sim 1.3\ cm^{-1}$, respectively.

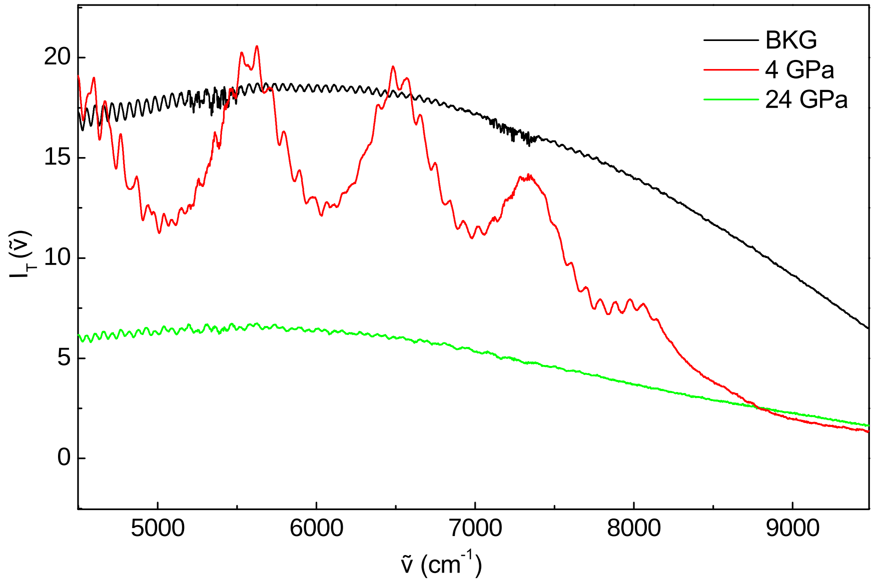

Figure S3: Spectra of the transmitted intensity collected on the background and on the 2nd sample at P=4 GPa and P=24 GPa.

3 - Extrapolation of the indirect gap in the NIR

As discussed in the manuscript, we estimated the indirect gap of our $MoTe_2$ samples as a function of pressure by means of a Tauc-plot extrapolation performed on the steep rise of the $A(\tilde{\nu})$ sigmoidal spectra after removal of the oscillating fringes through a standard Fourier Transform method.

We then define A_{min} and A_{max} as the average values of the low- and high-wavenumber plateau in the $A(\tilde{\nu})$ sigmoid (see Figs. S4 and S5). In order to coherently determine the fitting interval where a steep rise of the absorption occurs for all the considered spectra, we chose as the lower ($\tilde{\nu}_{low}$) and upper ($\tilde{\nu}_{up}$) limits of the wavenumber interval as follows:

$$\begin{cases} A(\tilde{\nu}_{low}) = A_{min} + 15\%(A_{max} - A_{min}) \\ A(\tilde{\nu}_{up}) = A_{min} + 85\%(A_{max} - A_{min}) \end{cases}$$

The adopted fitting ranges at selected pressures are shown in Figs. S4 and S5. We notice that, according to the theory, at low pressures the fitting range $[\tilde{\nu}_{low} - \tilde{\nu}_{up}]$ comprises a single, well-defined linear region in the Tauc plot. This can be conveniently fitted to estimate the indirect gap energy, as shown in Figs. S6(a) and S7(a). As the pressure increases, in the low-wavenumber side of the fitting interval, a sort of tail progressively shows up, preventing the possibility of a single linear fit in the whole region (see Figs. S6(b),(c), S7(b),(c).) The low-wavenumber *distortion* of the absorption spectrum can be ascribed to the onset of the so-called Urbach tail,[3–5] i.e. an exponential trend that appears in the absorbance curve near the band edge when localized states are present in the sample band structure, due to pressure-induced impurities or distortions in the crystal lattice.

The presence of the Urbach tail in the absorbance spectra at high pressure (approximately above 10 GPa) reduces the linear region available for the band gap estimate in the Tauc plot. Indeed, if we included in the Tauc plot extrapolation the low-wavenumber side of the interval, strongly affected by disorder effects, we would end up with a severe underestimation of the gap energy.

Based on the previous discussion, we proceeded with the Tauc plot extrapolation as follows. Below 10 GPa, we performed a single linear fit in the whole $\tilde{\nu}_{low} - \tilde{\nu}_{up}$ region. Above 10 GPa, we performed a double liner fit on two distinct sub-intervals: one at high wavenumbers associated with the band-gap related linear trend, in principle not affected by disorder effects, the other one at low wavenumbers where the trend is remarkably modified by the Urbach tail. Examples of single and double linear fits are shown in Figs. S6 and S7 at different pressure for both samples above and below 10 GPa. Band-gap estimates are reported in Fig. S8.

We notice that the high-wavenumber extrapolation is almost identical in the two samples (compare full and empty red circles in S8) and its trend connects almost continuously with the values obtained below 10 GPa (full and empty black circles in Fig. S8). The low-wavenumber extrapolation, on the other hand, gives us different gap estimates in different samples at

high pressure (compare full and empty blue circles in Fig. S8), suggesting the presence of sample/pressure dependent disorder effects. Moreover, these values significantly deviate from the trend observed at low pressures, where the gap extrapolation is more reliable.

On these bases, we can affirm that the energy gap reasonably goes to zero at the pressure value indicated by the high-wavenumber extrapolation (P∼24 GPa), while the P∼19 GPa value obtained through the low-wavenumber extrapolation represents an underestimation of the effective gap closing pressure. However, it is worth underlining that, even if we consider the P=19 GPa estimate, the band-gap closes at pressures significantly higher with respect to the metallization thresholds obtained through our FIR measurements (P=13-15 GPa) and through the resistivity measurements from the literature (P=10-13 GPa), validating our hypothesis on the two-steps metallization process.

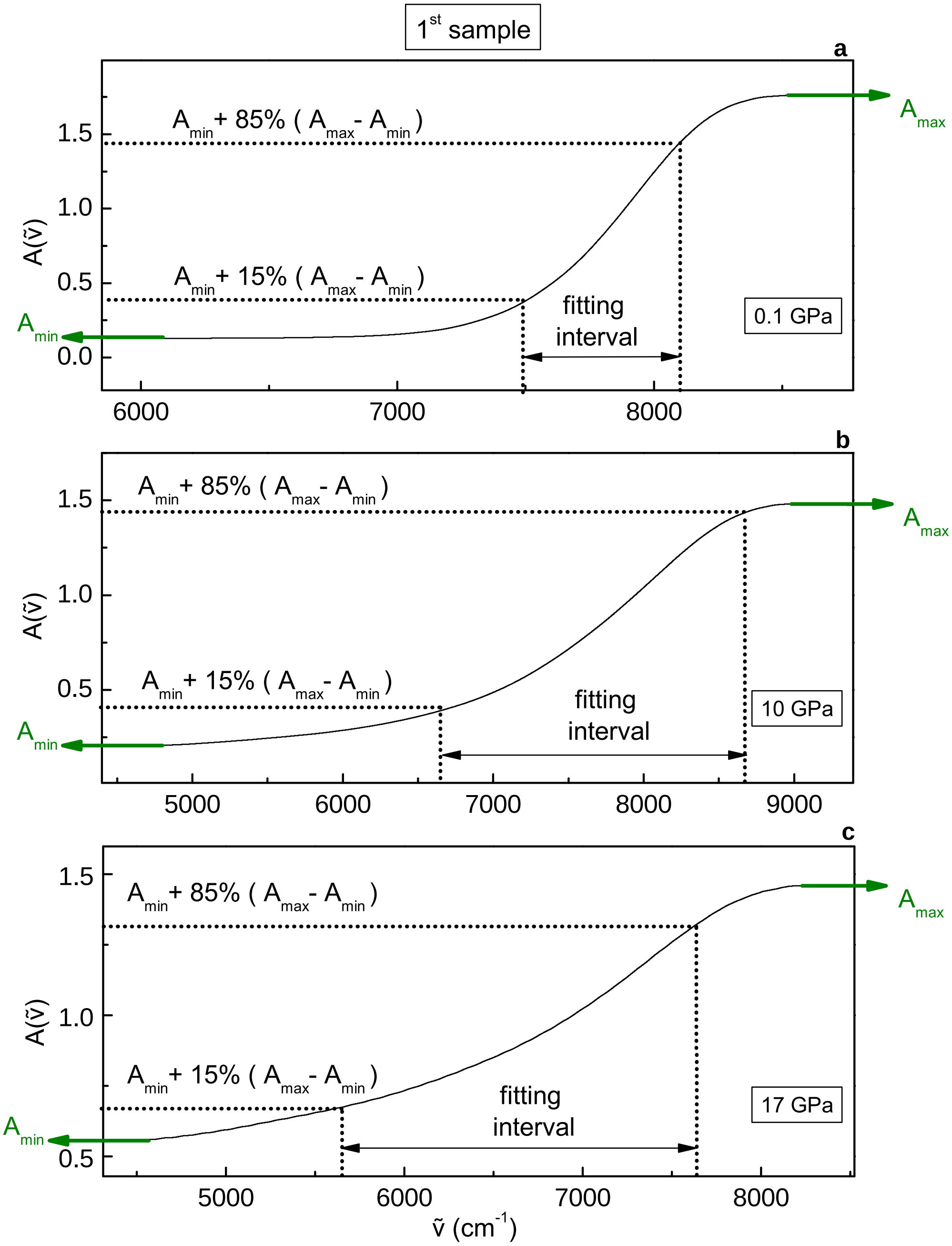

Figure S4: Definition of the fitting interval for the Tauc plot extrapolation at three representative pressures in the 1st sample.

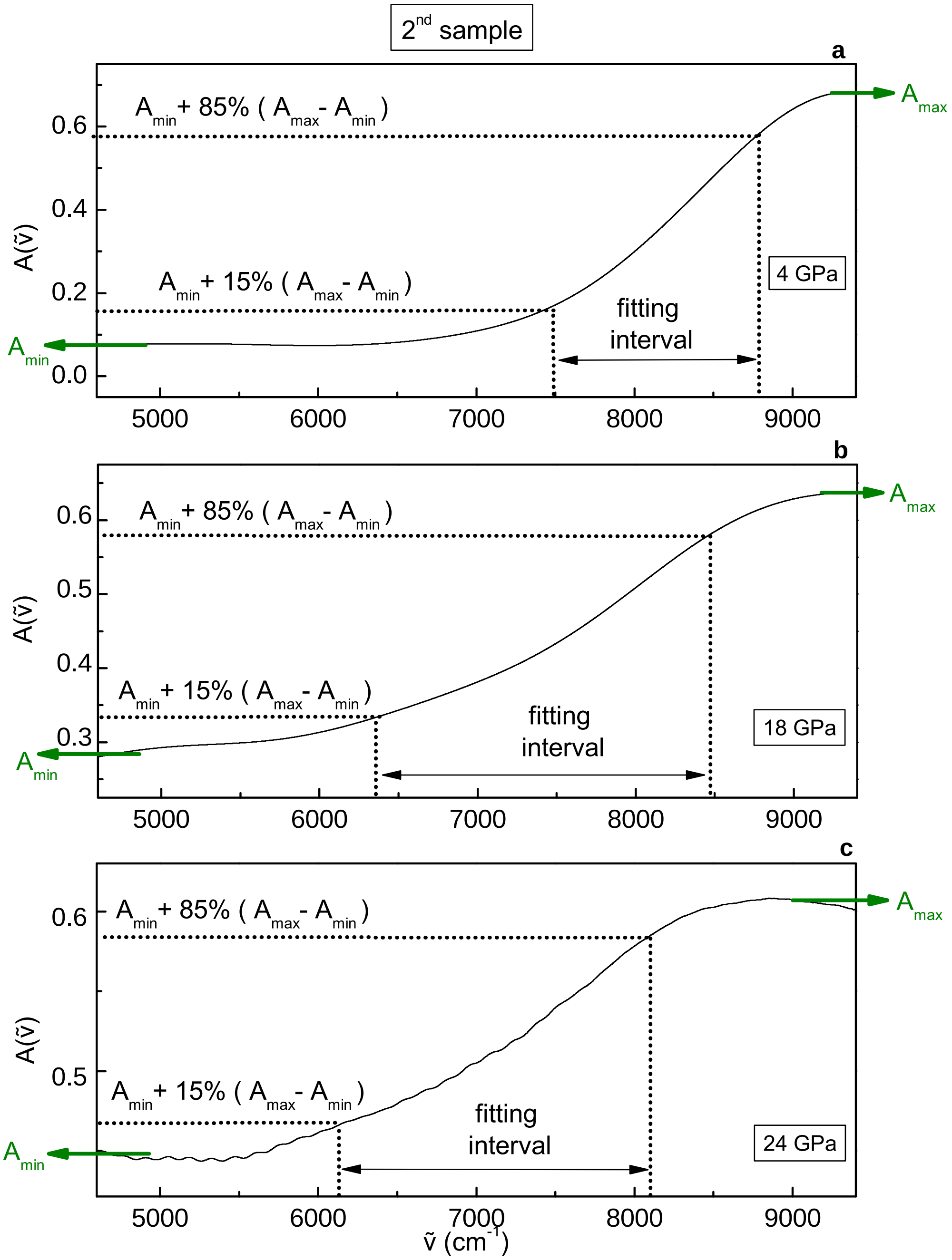

Figure S5: Definition of the fitting interval for the Tauc plot extrapolation at three representative pressures in the 2nd sample.

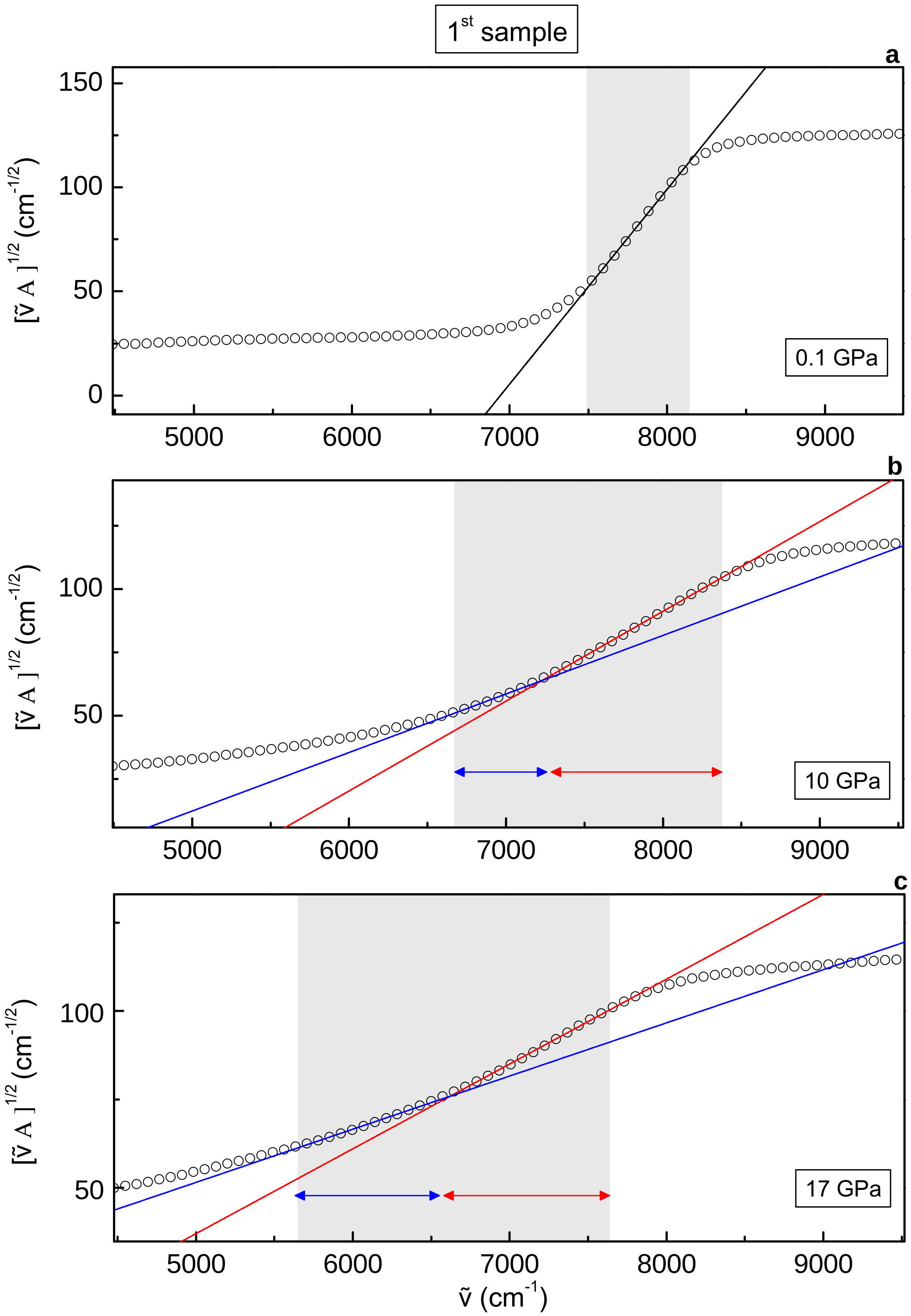

Figure S6: Tauc plot extrapolation of the indirect gap energy at three representative pressures in the 1st sample. The grey areas identify the fitting region defined through the procedure described in Fig. S4. At 0.1 GPa the $[\tilde{\nu} \cdot A]^{1/2}$ trend within the grey rectangle can be fitted by a single linear fit. At 10 GPa and 17 GPa a change of slope is evident within the interval making a double linear fit necessary: the blue (red) lines are obtained by fitting $[\tilde{\nu} \cdot A]^{1/2}$ in the region marked by the blue (red) arrows.

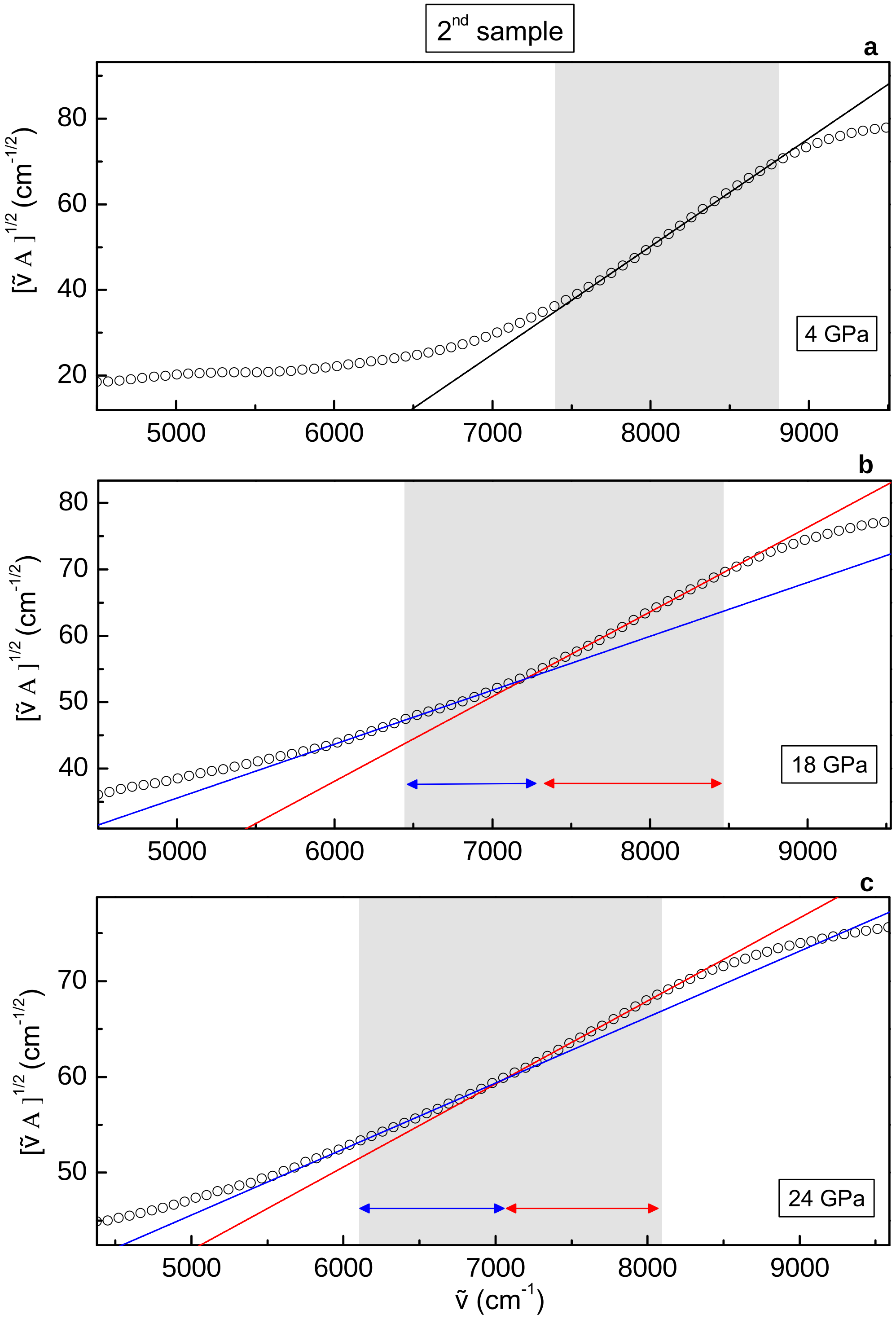

Figure S7: Tauc plot extrapolation of the indirect gap energy at three representative pressures in the 2nd sample. The grey areas identify the fitting region defined through the procedure described in Fig. S5. At 4 GPa the $[\tilde{\nu} \cdot A]^{1/2}$ trend within the grey rectangle can be fitted by a single linear fit. At 18 GPa and 24 GPa a change of slope is evident within the interval making a double linear fit necessary: the blue (red) lines are obtained by fitting $[\tilde{\nu} \cdot A]^{1/2}$ in the region marked by the blue (red) arrows.

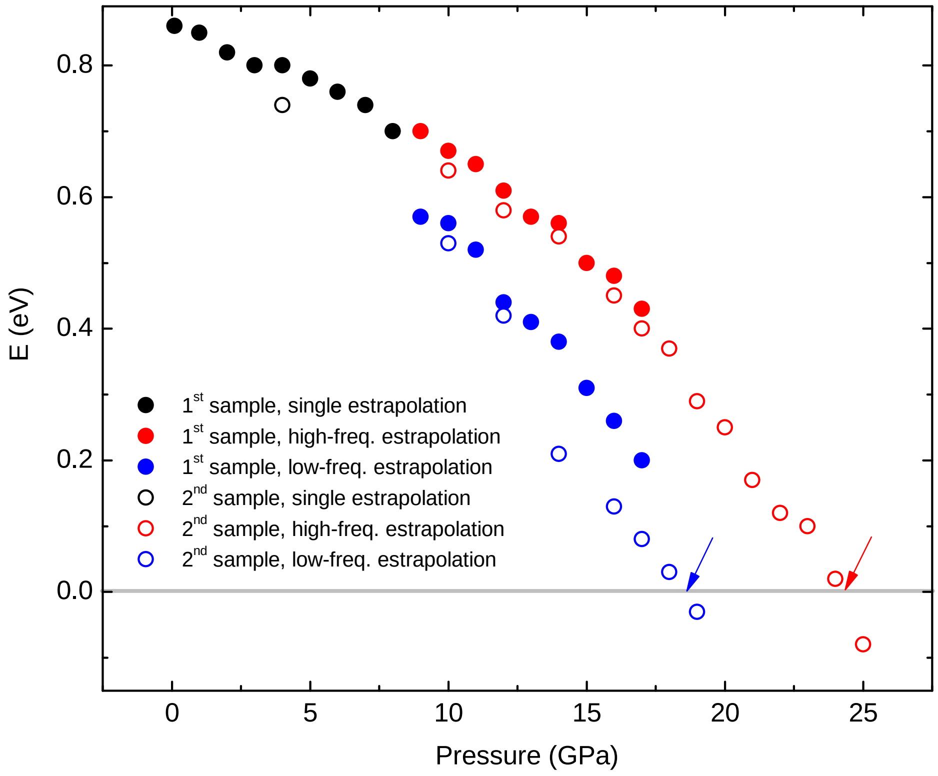

Figure S8: Gap values obtained through the Tauc plot extrapolation. Full and empty circles are associated with the 1st and 2nd sample respectively. Gap values obtained at low pressures (below 10 GPa) by a single linear fit are reported in black. Gap values obtained above 10 GPa through the high- (low-) wavenumber extrapolation are reported in red (blue). The red (blue) arrow indicates the gap closing pressure obtained through the high- (low-) extrapolation.

4 - Interference fringes in the FIR range

As explained before, absorbance spectra are defined as

$$A(\tilde{\nu}) = -log\frac{I_T^{Smpl}(\tilde{\nu})}{I_T^{Bkg}(\tilde{\nu})}$$

where, in this case, I_T^{Bkg} is the intensity transmitted by DAC filled with polyethylene only and I_T^{Smpl} is the intensity transmitted by the DAC with the $MoTe_2$ sample on top of polyethylene. The row spectra are shown in Fig. 2 of the main text.
Fig.S9(a),(b) shows I_T^{Bkg} and its Fourier transform FT[I_T^{Bkg}](t), respectively, in the FIR range. The FT[I_T^{Bkg}](t) spectrum exhibits a well-defined peak at $t \sim 4.7 \cdot 10^{-13}$ s, which can be identified with the Fourier Transform of a sinusoidal with period $\Omega \sim 2.1 \cdot 10^{12}$ s^{-1} $\sim$ 70 cm^{-1} (quite similar to $\Omega_1^{'}$, $\Omega_2^{'}$ values reported in section 2). Similarly to the previous discussion of the NIR data, this oscillation in the $I_T^{Bkg}(\tilde{\nu})$ spectrum can be attributed to interference effects due to multiple reflections of the incident beam between the diamond faces through the polyethylene medium.

In the calculation of the absorbance spectrum, we observed the presence of interference fringes that characterize $I_T^{Bkg}(\tilde{\nu})$ in each $A(\tilde{\nu})$ spectrum, their period and intensity being independent on the pressure evolution of the sample properties.
It is worth pointing out that, despite our best efforts, it was impossible to remove the fringes in the background spectrum in a reliable way due to reduced number of oscillation in the considered spectral range. Therefore, we decided to model the fringes through a least-square fitting procedure.

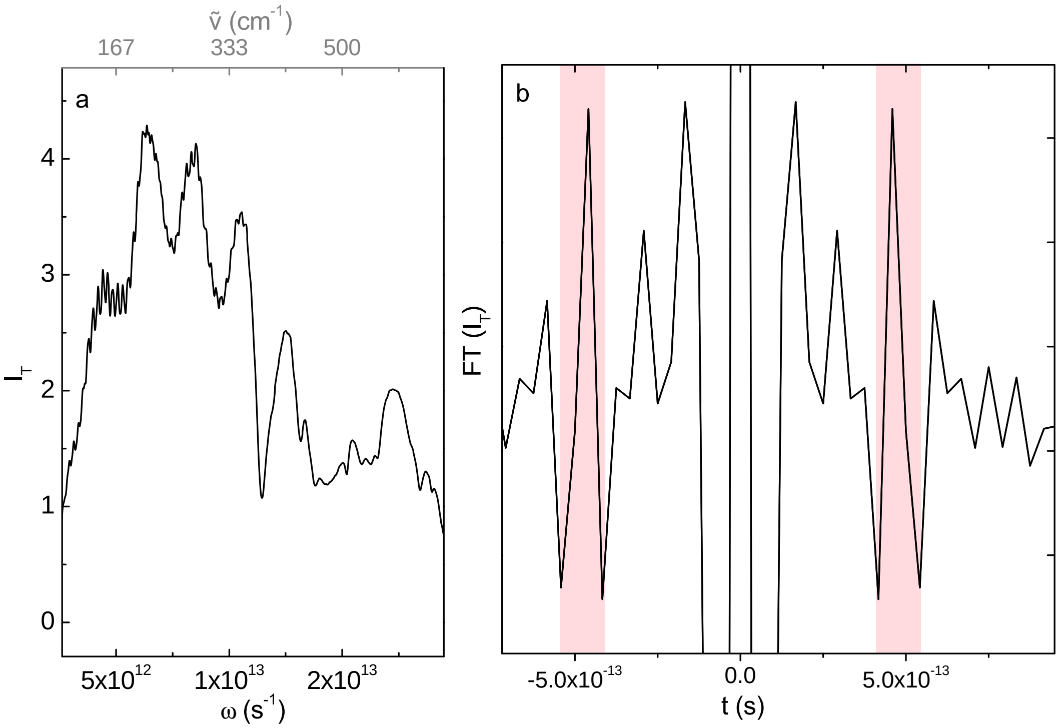

Figure S9: (a) Transmission spectrum collected on the background. The x-axis is reported both in [cm^{-1}] units (top) and in [s^{-1}] units (bottom) for an easier comparison with the Fourier Transform of the spectra. (b) Fourier Transform of the transmission spectrum shown in panel (a).

5 - Fitting procedure of the Fano peak

Fig. S10 shows the fitting procedure used to analyze the phonon profile at two representative pressures: 11 GPa (before the spectral weight increase) and 14 GPa (after the spectral weight increase). The sinusoidal fringes obtained in the fit maintain the same period on increasing pressure, while the steep dip on the high-wavenumber side of the phonon profile ($\sim$260 cm^{-1}) vanishes at 14 GPa, leading to the peak symmetrization already discussed in the main text.

As a further validation of the used fitting procedure, we make a comparison between the absorbance spectrum collected out of the cell, thus without the presence of interference fringes, and the absorbance spectrum at P=1 GPa obtained after the baseline (oscillation plus polynomial curve) subtraction, as reported in Fig. S11. Besides some slight differences in the peak width, intensity, and position, reasonably ascribable to the application of pressure, the profile of the two peaks is almost identical, confirming that the employed fitting procedure

does not alter the line-shape of the phonon.

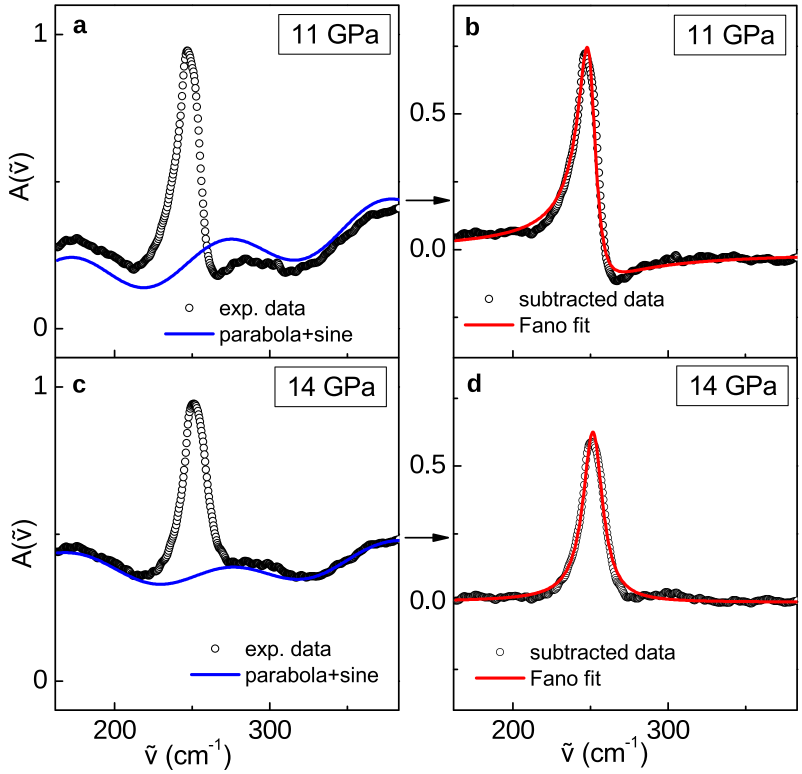

Figure S10: Absorbance spectra at 11 and 14 GPa before (a and c panels) and after (b and d panels) the baseline (sinusoidal plus parabolic function) subtraction. Black dots represent the experimental data, while the continuous lines represent the fitting functions: blue for the background and red for the Fano curve. Notice that at 11 GPa (a) the experimental data deceptively appear to be under/overestimated by the baseline at values below/above the phonon peak central wavenumber. This is a direct consequence of the Fano function behavior outside the peak region, as shown in panel b.

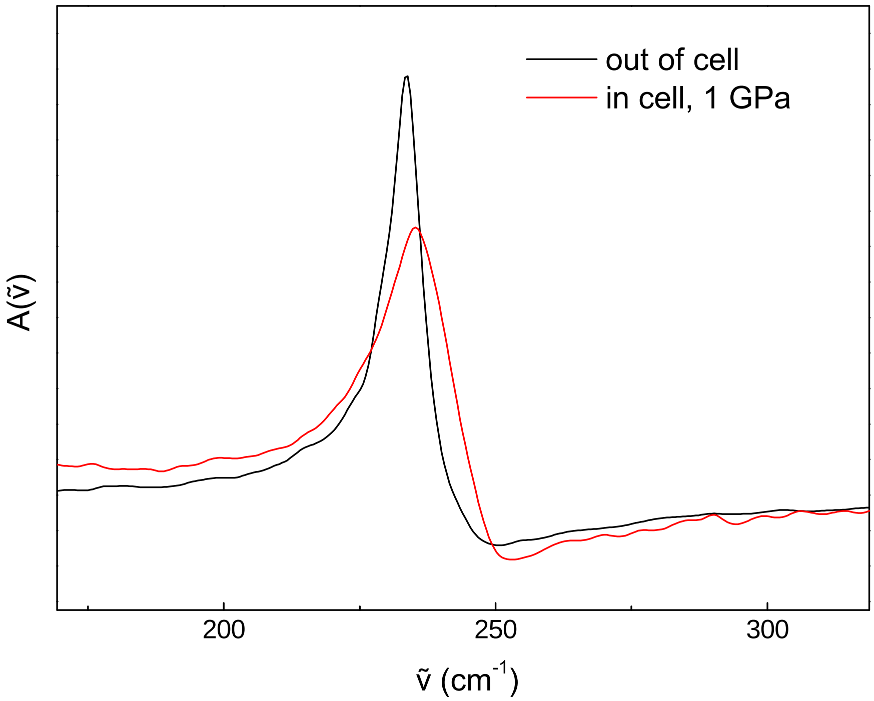

Figure S11: Absorbance spectra collected out of the cell (black curve) and in the cell at 1 GPa (red curve). The latter was obtained after subtracting a sinusoidal and a parabolic curve to the row data, as explained in the main text.

6 - Reversibility

The pressure-induced metallization that we observed for $MoTe_2$ is, substantially, a reversible process. This means that the pressure-induced modification of the electronic properties in MoTe2 is not permanent and the original electronic properties can be recovered once the sample is brought back to ambient conditions. In the FIR measurements, in figure S12 (a), we can see that, after the increase in the spectral weight observed above 13 GPa, the spectrum collected at $\sim$0.5 GPa during the decompression run displays an average absorption level comparable to that obtained before the compression. Moreover, the E_{1u} phonon peak, which was screened above 13 GPa, turns visible again as the pressure is lowered. It is worth noticing that the intensity of the phonon mode at 3 GPa after decompression is slightly lower than that of the peak collected at 3 GPa during the compression cycle. This could be related to a small reduction in the crystal quality after the application of high pressure. To sum up, we can say that the metallic behaviour induced by the pressure-induced reduction

in the energy separation between doping levels and conduction band can be reversed once the pressure on the sample is released, although the lattice disorder partially increases. The same holds for NIR measurements, as shown in figure S12 (b). The results we obtained are coherent with the work of Yang et al.,[6] which observed a reversible behavior in bulk $MoTe_2$ under the application of hydrostatic pressure up to ∼30 GPa.

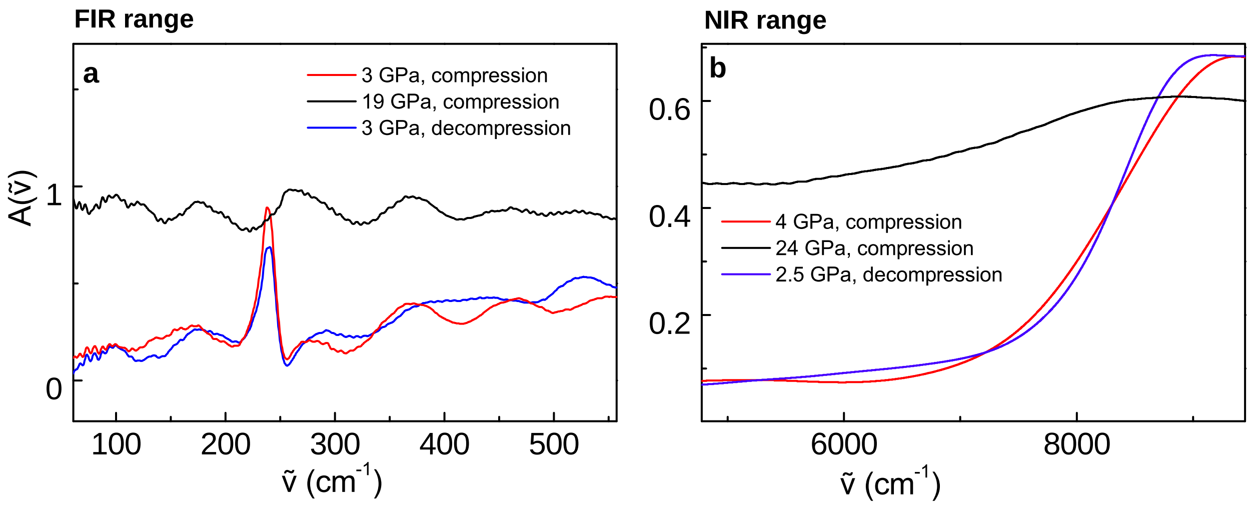

Figure S12: Comparison between the absorbance spectra collected during the compression (red and black curves) and decompression (blue curve) runs. Panel (a) FIR spectra, panel (b) NIR spectra.

References

(1) Li, H. H. *Journal of Physical and Chemical Reference Data* **1976**, *5*, 329–528.

(2) Grasso, V.; Mondio, G.; Saitta, G. *Journal of Physics C: Solid State Physics* **1972**, *5*, 1101–1108.

(3) Bhatt, R.; Bhaumik, I.; Ganesamoorthy, S.; Karnal, A. K.; Swami, M. K.; Patel, H. S.; Gupta, P. K. *physica status solidi (a)* **2011**, *209*, 176–180.

(4) **2013**, *113*.

(5) Buontempo, U.; Degiorgi, E.; Postorino, P.; Nardone, M. *Physical Review B* **1995**, *52*, 874–878.

(6) Yang, L.; Dai, L.; Li, H.; Hu, H.; Liu, K.; Pu, C.; Hong, M.; Liu, P. Characterization of the pressure-induced phase transition of metallization for $MoTe_2$ under hydrostatic and non-hydrostatic conditions. *AIP Adv.* **2019**, *9*, 065104.
